# Do High Psychopaths Care More about Moral Consequences than Low Psychopaths in Chinese Culture? An Exploration Using the CNI Model

**DOI:** 10.3390/healthcare8040505

**Published:** 2020-11-21

**Authors:** Shenglan Li, Daoqun Ding, Zhihui Wu, Liangliang Yi, Ji Lai, Le Dang

**Affiliations:** 1 Department of Psychology, School of Education Science, Hunan Normal University, Changsha 410081, China; shenglan198989@163.com (S.L.); oc42066@163.com (Z.W.); liang@hnust.edu.cn (L.Y.); susan_mili@126.com (J.L.); 2 Cognition and Human Behavior Key Laboratory of Hunan Province, Hunan Normal University, Changsha 410081, China; 3 Normal College, Hunan University of Arts and Science, Changde 415000, China; 4 Department of Psychology, Faculty of Social Sciences, University of Macau, Macau 999078, China; YB97304@connect.umac.mo

**Keywords:** psychopathy, moral judgment, deontological, utilitarian, CNI model

## Abstract

*Purpose:* Fewer studies are about the influence of psychopath traits on moral judgment and the underlying psychological mechanism in Chinese cultural background. In this paper, we use the creative CNI (Consequences, Norms, Inaction versus action) model to quantify the subject’s reaction to moral dilemmas. *Method:* In this research, the Chinese version of the Levenson Psychopathic Scale, CNI model materials, and a multinomial model to further analyze the associations among the psychopathy characteristics and utilitarian moral judgment are applied. The CNI model is proposed by Gawronski et al., which can quantify the subjects’ sensitivity to moral consequence, sensitivity to moral norms, and the general preference for inaction or action in moral dilemmas. *Result:* This study finds that there were significant differences in the utilitarian moral judgment between the groups, *t* (360) = 3.24, *p* = 0.001, and Cohen’s *d* = 0.36. The analysis results of the CNI model show that the high psychopathy group on the *N* parameter was significantly lower than the group of low psychopathy, ΔG^2^ (2) = 79.70, *p* = 0.001. In terms of the *C* parameter, we found no significant distinctions between the two groups, ΔG^2^ (2) = 1.356, *p* = 0.244. For the *I* parameter, the two groups also have no significant differences, ΔG^2^ (2) = 0.093, *p* = 0.76. *Conclusion:* Persons with high psychopathy traits prefer to make more utilitarian moral judgments and have a weak sensitivity to moral norms (*N*). The sensitivity to consequences (*C*) of the two groups is no significant difference. The general preference for inaction versus action (*I*) also has no significant differences between those two groups. Moreover, the CNI model fits well in Chinese subjects.

## 1. Introduction

The concept of psychopathy originates from Theophrastus’s description of the unscrupulous man who is a slippery, pathological liar and who does not feel guilt or self-reproach for his actions [1]. Psychopathic individuals are characterized by high impulsivity and aggressiveness, a lack of guilt or remorse for their immoral behaviors, and an inability to learn from punishment [2,3]. At present, the most widely accepted definition of psychopathy was proposed by Professor Hare, a Canadian psychiatrist. He defines this type of personality disorder as a combination of interpersonal relationships, emotional experience, lifestyle, antisocial characteristics, and immoral behavior performance [2]. These interpersonal and emotional characteristics cause psychopaths to behave in ways that deviate from social rules, such as impulsive and dishonest behaviors (not necessarily criminal behavior) [2,4]. Most of the psychopathic personality defects developed by teenagers will continue into adulthood [5]. Furthermore, recent studies show that psychopathic traits are also distributed in the subclinical population [6,7]. Instead of merely categorizing people as psychopaths and non-psychopaths, psychopathy is reflected in the degree of difference, which can be found in the general public by measuring the level of psychopathic traits [8,9].

The assessment tools of psychopathy mainly include a checklist and self-report scales. Hare developed and compiled a psychopathy checklist (PCL) for the screening of psychopathy [10,11,12,13]. The PCL-R scale has excellent validity and reliability [14]. Nonetheless, the PCL-R scale is difficult to use in non-criminal groups. With the deepening of studies concerning psychopathy traits, the subjects of study have gradually been extended to the subclinical population [11]. Consequently, researchers have revised several self-report scales to screen for psychopathic traits in ordinary people [15]. Through self-report scale screening, we can divide such subjects into high and low psychopathy groups, according to their levels of psychopathic traits. Among the scales, the Levenson Psychopathy Scale is the shortest, with only 26 items, and a large number of studies have confirmed that the scale has excellent reliability and validity in several samples, including an incarcerated population, general community samples, and children or adolescents. It has also shown excellent cross-cultural advantages, having excellent reliability and validity in Chinese populations [16,17,18,19].

Early researchers put forward the concept of “born criminal” and labeled psychopaths as morally despicable [7]. However, there is little research on the moral judgment of the psychopath, especially in Chinese culture and society. The definition of moral judgment is the evaluation of a person’s quality or specific behavior according to the virtues that culture and subculture stipulate that an individual must possess [18,19]. An important paradigm of moral judgment is the moral dilemma, a valuable tool for studying which factors trigger the potential psychological processes of human moral cognition [18]. One principle is conflicted with another in a moral dilemma; the most prominent of which is the trolley dilemma [20,21]. This dilemma describes the following scene: a runaway train is rapidly approaching a fork in the track. There are five railway workers on the left track, but there is only one railway worker on the right track. If you do nothing, the train will go left, killing five workers. The only way to avoid the death of these workers is to throw the switch, which will cause the train to move to the right, killing the one worker. To avoid the death of five workers, should you pull the switch? Individuals are aware of the incompatibility of the two choices and the different consequences. After reading the story, the subjects were asked to choose whether they should take action, i.e., throw the switch, thus making what is usually called a moral judgment [18,19].

The most influential theory in moral judgment is the dual process theory [22]. The dual process theory holds that moral judgment requires the participation of consciousness and rational reasoning and consumes the cognitive resources of individuals. However, it also acknowledges that automatic, intuitive emotions, which consume fewer cognitive resources, also play a part in moral judgment. Parts of the process of individual moral judgment, rational cognition, and emotional intuition are in a competitive relationship. Early studies of moral cognition divided subjective decisions into utilitarian and deontological judgments [18]. Deontological judgments are thought to be rapid, emotional, and resource-independent, while utilitarian judgments are thought to be slow, cognitive, and effortful. According to deontology, the lives of five people cannot be regarded as more important than that of one person. It is a moral duty not only not to kill people but also to save people. Deontology thus requires that individuals should not act in the face of such a dilemma. According to the tenets of deontology, the morality of behavior depends on the consistency of the behavior with moral norms. By contrast, according to utilitarian judgment, since five is greater than one, five lives are more important than one life. When choosing who must be abandoned, the lives of the few should be sacrificed to save the lives of the many. It is a tenet of utilitarianism that the morality of a behavior depends on the effect of that behavior on overall well-being [19,20]. However, the theory does not consider the possibility that subjects may be driven by two motives when making moral decisions. In traditional moral dilemmas, an individual who takes action is considered to have made a utilitarian moral judgment. In contrast, one who has chosen not to take action is considered to have made a deontological judgment. The conceptual meaning of individual choice is ambiguous because moral consequences and norms are not manipulated in terms of the definitions of utilitarianism and deontology [21,22].

Traditional methods regard deontology and utilitarianism as negatively correlated and confuse explicit moral judgments with intrinsic moral tendencies. Many researchers believe that deontological judgment and utilitarian judgment are the results of two distinct psychological processes [19,20]. Therefore, Conway et al. proposed the process-dissociation approach (PD) [23]. The process-dissociation (PD) approach offers a way of overcoming this limitation and quantifying the deontological and utilitarian tendencies of individuals. The core idea of the PD approach is the difference between incongruent dilemmas, in which deontological and utilitarian psychological processes lead to different responses, and congruent dilemmas, in which the two psychological processes lead to the same response. The congruent dilemma and the incongruent dilemma in the PD approach involve only the prohibited dilemmas. In the incongruent dilemmas, the benefit of the action is greater than the loss, while in the congruent dilemma, the benefit of the action is less than the loss. By comparing the reactions of a consistent process with the reactions of a competitive process, we can quantify the relative effects of each psychological process algebraically. *U* parameter is calculated by taking the difference in the proportion of “unacceptable” responses between the incongruent and congruent dilemmas: *U* = *p* (unacceptable/congruent) − *p* (unacceptable/incongruent). *D* parameter is calculated as the proportion of “unacceptable” responses in incongruent dilemmas relative to all non-utilitarian responses: *D* = *p* (unacceptable/incongruent)/(1-*U*).

These two formulas provide a way of quantifying the strength of a subject’s deontological and utilitarian tendencies [23]. However, the PD approach confuses the deontological and utilitarian tendencies of the subject with the subject’s reaction preference, which it cannot examine. Traditional methods and the PD approach are only applicable to dilemmas relating to proscriptive norms. By contrast, the CNI (Consequences, Norms, Inaction versus action) model applies to dilemmas relating to either proscriptive or prescriptive norms and allows independent quantification of the participant’s sensitivity to both moral consequences and moral norms or their general preference for inaction or action.

To address the limitations of the previous two approaches, Gawronski and his colleagues (2017) constructed the CNI model. They used the concept of a multinomial processing tree (MPT) in an attempt to separate the different cognitive processes of subjects making moral decisions when faced with dilemmas [21,24]. According to the CNI model, utilitarian judgments originate from sensitivity to morally relevant consequences, and deontological judgments originate from sensitivity to moral norms. This model divides moral judgment into three psychological processes: sensitivity to consequences (parameter *C*), sensitivity to moral norms (parameter *N*), and the general preference for inaction versus action (parameter *I*). The three parameters represent the three underlying psychological processes behind moral decisions: utilitarian judgment, deontological judgment, and reaction tendency. Thus, the CNI model quantifies these three different psychological processes [21,24].

**Figure 1:**
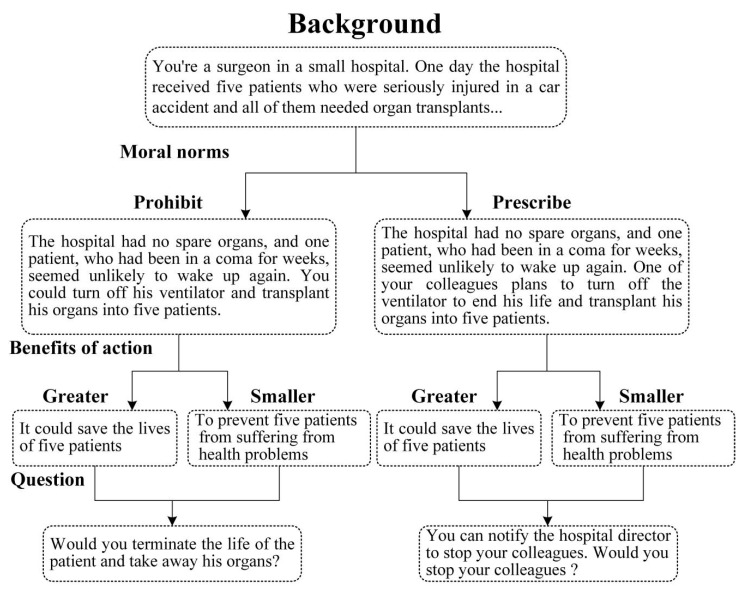
An example of the CNI (Consequences, Norms, Inaction versus action) model moral dilemma material.

According to the CNI model, moral judgments can be divided into two broad categories. The first category is utilitarian judgment, which requires individuals to be sensitive to moral consequences and to conduct experimental operations on the results in moral dilemmas. The second type is deontology judgment, which requires the individual to be sensitive to moral norms, to conduct experimental operations based on moral norms in moral dilemmas. To verify whether the CNI model is suitable for moral dilemma research and can suitably separate potential psychological processes, Gawronski et al., (2017) designed six basic moral dilemma scenarios [25]. Each moral dilemma includes four versions of the benefit/cost ratio of action (benefit greater than cost vs. benefit less than cost) × moral norms (inhibition action vs. promotion action) (see the example in Figure 1). In these 24 versions of the moral dilemma, the subjects need to decide to take action or not. In addition, the four types of dilemmas stem from real moral events in the real world, which easily stimulate a broad moral discussion. Furthermore, the CNI model uses the subjects’ behavior data to fit the established model and to estimate the probability of each psychological process [24]. Data fitting can be carried out employing various statistical methods, for example, by using the goodness of fit statistic G^2^. If the G^2^ value is not significant, it indicates that the model and data fit well, while if the G^2^ value is significant, it indicates that the model and data do not fit well [26]. Another method is to set the parameters of a certain psychological process to 0 or 0.5 according to other standards, and then evaluate the influence of model parameters on model fit [24]. This produces a significant statistical distinction between the actual observation probability and the model prediction probability.

Some Western studies based on the traditional moral dilemma method show that high psychopaths are more willing than low psychopaths to accept behaviors that hurt others and are more inclined to make utilitarian moral judgments rather than deontological judgments [27,28,29,30,31,32,33]. In contrast, Cima et al. suggest that the moral judgments of psychopaths are not significantly different from those of the general population [34]. Few studies have been conducted on psychopathy in Chinese subjects, especially in the field of moral judgment, and there has been a lack of consistency in past research on whether psychopaths make more utilitarian moral judgments. Thus, whether persons with high levels of psychopathy will make more utilitarian moral judgments needs further verification in Chinese subjects. The underlying mechanism behind the utilitarian moral judgment of high psychopathic individuals is also worthy of further study in Chinese culture.

The samples in previous research included prison inmates, college students, and adult communities [27,28,29,30,31,32,33,34]. Several previous studies on this subject focused on the psychopathic traits of prison inmates and examined differences in the utilitarian moral judgments of prison inmates and non-incarcerated subjects. However, the particularity of the prison environment and the difference in education level between the two groups may have affected the differences in moral judgment between the groups. By contrast, we conducted our study with university students since, in subclinical undergraduate subjects, the influence of psychopathic traits on utilitarian moral judgments is less affected by additional variables. We used the Levenson Self-Report Psychopathy Scale (LSRP), which we deemed suitable for assessing undergraduate subjects.

In Western culture, some research has found that high psychopaths will make more utilitarian moral judgments [27,28,29,30,31,32,33]. We believe it non-intuitive to infer from the premise of utilitarian moral judgments that high psychopaths are more concerned with morally relevant consequences and with the interests of the majority [24,27,28]. These results also do not accord with the behavioral characteristics of psychopaths, who seem more than willing to accept behavior that hurts others regardless of the interests of the majority. The traditional method fails to capture the differences between the two situations [24]. The CNI model may provide a more in-depth explanation of the psychological mechanism of moral judgment in Chinese subjects. While previous methods have not been able to distinguish whether the aforementioned differences in behavior were due to individual differences in utilitarian or deontological tendencies or to preferences for action or inaction, the CNI model distinguishes between these psychological processes.

Moral judgment is also greatly influenced by cultural differences. The CNI model is a new moral judgment model proposed by Western scholars, and the moral dilemma materials are compiled on the background of Western culture. Hence, moral dilemma materials applicable to China were worth further exploration, whereas whether the CNI model is suitable for Chinese subjects needs further verification. Using the CNI model to explore the relationship between psychopathy and moral judgment, Gawronski et al. (2017) conducted two replication studies on Western subjects. The first study found a significant difference between the high and low psychopathy groups in the *N* parameter but no significant differences for the *C* and *I* parameters. However, the results of the second study revealed significant differences between the two groups for all three parameters. It is worth further exploring the similarities and differences between the results in Chinese subjects. We hypothesized that persons with high psychopathy traits have a preference for utilitarian moral judgments and a weak sensitivity to moral norms (*N*). Our research verifies the reliability and applicability of the CNI model, which offers a useful framework for exploring the psychological processes behind moral judgments in the context of Chinese culture.

## 2. Materials and Methods

### 2.1. Participants and Procedure

Using a simple random sampling method, we recruited 900 students from three colleges in Hunan Province in China, 31 of whom failed to complete all the tests. Thus, we obtained a final valid sample of 869 students, of whom 282 were men and 587 women. The average age was 19.03 ± 1.71, and 97.7% of the subjects were undergraduates. Using G* Power software, we determined that the sample size of 869 in this study would provide at least 99% statistical efficacy [35]. The sample size of 869 in this study could provide at least 99% statistical efficacy. Subjects were tested online and had not taken a similar moral judgment test before taking part in the study. Participants in this research were right-handed and had no psychiatric history. The exclusion criteria were (i) significant cognitive impairment and history of mental illness; (ii) previous participation in similar moral judgment studies; or (iii) an inability to complete all measurements. All subjects agreed to participate after receiving written and oral explanations of the purpose of the study, and all of them signed an informed consent form. The assessment was carried out individually, and the participants received a small gift after completing the assessment. The research was conducted according to the Declaration of Helsinki.

Although the term psychopathy was originally used to describe a legally-determined group of criminals, psychopathic traits can also be found in subclinical groups. In ordinary subjects, psychopathic traits exist in different degrees [36,37]. According to the grouping method in Gawronskia and Conway et al.’s study, subjects were grouped according to their percentage of a high or low score on the screening scale [24]. In this study, the subjects were subclinical college students, and the LSRP for the screening of psychopathy traits was used to classify the high psychopathy group and the low psychopathy group according to the subjects’ scores. The subjects were divided into high and low psychopathy groups to conduct further model fitting to investigate the differences between the two groups. This grouping method has been used in previous studies and can greatly simplify the statistical analysis while making the interpretation and expression of the results more straightforward [38]. The CNI model quantifies individuals’ sensitivity to consequences, sensitivity to norms, and the general preference for inaction and action and can only output the parameter values of each group of subjects. According to the model’s analysis method, the differences in the three parameters between the high psychopathy group and the low psychopathy group are visually compared. Using the Levenson Self-Report Psychopathy Scale (LSRP), the first 20% of participants were assigned to the high level of psychopathic traits group, and the final 20% of subjects were placed in the low level of psychopathic traits group. A total of 174 participants had high psychopathic traits (*M* = 84.21, *SD* = 6.39), of which 78 were male. Their mean age was 19.39 ± 2.67. A total of 188 subjects (*M* = 47.44, *SD* = 6.28) were in the lower psychopathic traits group, including 53 males, with an average age of 18.94 ± 1.37. There were significant differences in the degree of psychopathic characteristics across the two groups in this study: *t* = 56.18, *p* = 0.00, and Cohen’s *d* = 5.80.

### 2.2. Research Material

#### 2.2.1. The Levenson Self-Report Psychopathy Scale (LSRP)

For this research, the Chinese version of the LSRP was used, which includes 26 questions, measuring two dimensions: primary psychopathy and secondary psychopathy. Primary psychopathy is usually influenced by cognitive, emotional, and biological genetic factors and is generally associated with callousness, interpersonal manipulation, and selfishness. In clinical studies, secondary psychopathology is considered an emotional disturbance or internal conflict that leads to antisocial or violent behavior [8,9]. The LSRP uses a Likert 6-point scale: 1 = completely disagree, 2 = disagree, 3 = a little disagree, 4 = somewhat agree, 5 = agree, and 6 = completely agree. The internal consistency coefficient of the scale in this research was 0.85. The internal consistency coefficients of the primary psychopathy and secondary psychopathy subscales were 0.85 and 0.74.

#### 2.2.2. CNI Model Moral Judgment Materials

For our study, we used 24 moral dilemmas from a paper written by Gawronski et al. (2017), which had been translated into Chinese. The content and structure of the Chinese version of the CNI model are the same as those of the English version. The experiment materials comprised 12 dilemmas which norms prohibit and 12 dilemmas which norms permit. When the subjects confront a dilemma concerning a norm prohibition, the moral norm inhibits action. On the contrary, in the face of a dilemma that a moral norm permits, the moral norms in the dilemma promote action. Both the norm prohibit dilemmas and the norm permit dilemmas contain two situations, one is that the benefit of the action is greater than the loss, the other is that benefit of the action is smaller than the loss. Therefore, the CNI model contains four different types of dilemmas. These 24 moral dilemmas were randomly presented to the subjects, who were asked to make a judgment. In each dilemma, the subject is asked whether he or she would accept the described behavior or not. Unlike previous studies of moral judgment, the materials used not only norms prohibited moral dilemmas but also norms permitted moral dilemmas. Therefore, our study can comprehensively explain different moral judgments and thus can further explain the psychological mechanisms behind moral judgments. In Figure 1, we give an example of the CNI moral dilemma material.

## 3. Results

### 3.1. Descriptive Statistics

The descriptive statistics and Pearson correlation for each variable are presented in Table 1. This study finds that psychopathy is significantly positively correlated with traditional utilitarianism judgments (*r* = 0.12, *p* = 0.000). The primary psychopathy is significantly positively correlated with traditional utilitarianism judgments (*r* = 0.09, *p* = 0.004), and the secondary psychopathy is significantly positively correlated with traditional utilitarianism moral judgments (*r* = 0.11, *p* = 0.001). The *D* coefficient of deontological tendency is significantly positive relating to traditional utilitarian moral judgment (*r* = −0.76, *p* = 0.000), and the *U* coefficient of utilitarian tendency is significantly positively correlated with traditional utilitarianism moral judgment *(r* = 0.53, *p* = 0.000). These findings provide preliminary support for the subsequent data analysis.

### 3.2. The High Psychopathy and Low Psychopathy Groups Analysis Results in Four Moral Dilemmas

In the LSRP, the score identifying the low psychopathic traits group was 55, while for the high psychopathic traits group, it was 77. There were significant differences in the levels of psychopathic traits among those two groups, *t* = 56.18, *p* = 0.00. The high and low psychopathic traits groups mean and standard deviation of distinctive moral dilemmas responses are shown in Table 2.

### 3.3. Traditional Moral Dilemma Analysis Results

As with traditional moral dilemma research methods, we first examined subjects’ responses to moral dilemmas in which the interests of the individual action outweigh the losses. In conventional research methods, the preference of individuals for action over inaction in such dilemmas is interpreted as a preference for utilitarian judgment over deontological judgment. In this dilemma, the high psychopathic traits group subjects were more inclined to take action than to do nothing than the subjects in the low psychopathic traits group. Moreover, there was a significant distinction between the two groups, *t* (362) = 3.24, *p* = 0.001, and Cohen’s *d* = 0.36. Consistent with the results of formal study, people with high psychopathic trait preferences tend to make more utilitarian moral judgments.

### 3.4. The Process Dissociation Technique Analysis

The process dissociation technique can be thought of as a simple MPT model with only two cognitive parameters. The process dissociation technique is used to measure the intensity of utilitarianism and deontological tendencies [29]. The process dissociation technique considers the utilitarian tendency (*U* parameter) and the deontological tendency (*D* parameter) to be independent of each other. The 12 prohibited moral dilemmas in the CNI model research material, the incongruent dilemma is that the benefit of the action is greater than the loss of the action, and the congruent dilemma is that the benefit of the action is less than the loss of the action. The experimental results further show that there exists no significant difference in the *U* parameter between the subjects in the high psychopathic traits (*M* = 0.14, *SD* = 0.23) and the low psychopathic traits groups (*M* = 0.15, *SD* = 0.23), *t* (362) = 0.56, *p* = 0.578, and Cohen’s *d* = 0.043. The subjects in the high psychopathic traits group (*M* = 0.59, *SD* = 0.23) and the subjects in the low psychopathic traits group had significant differences in the *D* parameter (*M* = 0.69, *SD* = 0.23), *t* (362) = 4.28, *p* = 0.000, and Cohen’s *d*= 0.44.

### 3.5. CNI Moral Model Analysis Results

The software multiTree was used in the CNI model analyses [24]. Without considering the subjects’ gender, the CNI model data for the total subjects fit well ΔG^2^ (1) = 1.52, *p* = 0.22. The *C* parameters (*M* = 0.15, 95%CI (0.132, 0.158)) were significantly higher than 0, ΔG^2^ (1) = 471.74, *p* < 0.001, and the *N* parameters (*M* = 0.30, 95%CI (0.284, 0.315)) were also significantly higher than 0, ΔG^2^ (1) = 1470.94, *p* < 0.001. The results suggest that all the subjects are highly sensitive to both moral consequences and norms when faced with a moral dilemma. The *I* parameter deviates significantly from its reference point of 0.5, ΔG^2^ (1) =7.01, *p* < 0.01, which indicates that the response distribution of action and inaction are not equal in all samples and that the Chinese subjects tend to choose an action in all samples. The influence of gender on the moral judgment was further investigated, and the results of model fitting showed that there were no significant differences between male and female subjects in the three parameters, *p* > 0.01.

**Figure 2:**
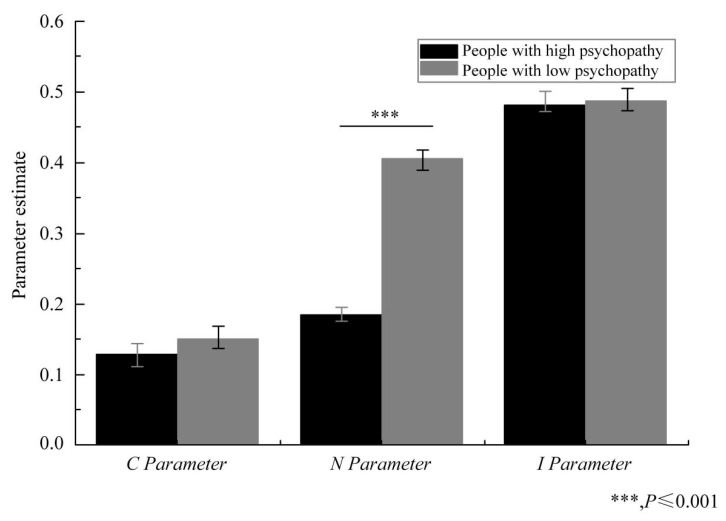
The CNI model’s high and low psychopathy groups’ parameter estimates for sensitivity to moral consequences (*C*), sensitivity to moral norms (*N*), and the general preference for inaction versus action (*I*).

In the high psychopathy and low psychopathy groups, the CNI model data also fit well, ΔG^2^ (2) = 0.493 and *p* = 0.781. The score of the subjects with high psychopathy on the *N* parameter was significantly lower than that of the subjects with low psychopathy, ΔG^2^ (2) = 79.70, and *p* < 0.001. In terms of the *C* parameter, there was no significant difference among the subjects in the high psychopathy and low psychopathy groups, ΔG^2^ (2) = 1.356 and *p* = 0.244. For the *I* parameter, there was also no substantial difference among the two groups, ΔG^2^ (2) = 0.093 and *p* = 0.76 (see Figure 2). Specifically, there was no significant difference between the subjects with high psychopathic traits and the participants with low psychopathic traits in the sensitivity to moral consequences (*C*), nor was there a substantial difference in their general preference for inaction versus action. In summary, these results indicate: (1) Persons with high psychopathy have a low sensitivity to moral norms. (2) There was not a substantial difference in sensitivity to moral consequences between those subjects with high psychopathic traits and those with low psychopathic traits. (3) The group with high psychopathic traits’ general preference for inaction versus action, was similar to that of the group with low psychopathic traits.

## 4. Discussion

In this study, we demonstrate that people with high psychopathic tendencies tend to make more utilitarian judgments in moral dilemmas in Chinese culture. This finding is consistent with the results of Bartels and Yu Gao et al. [27,28,29,30,31,32,33], but are different from those of Cima [34]. Furthermore, we found that the high psychopathy group scored significantly lower on deontological tendency (*D*) than did the low psychopathy group. However, the utilitarianism tendency (*U*) of the two groups is not significantly different. The affective deficits of people with high psychopathic traits can affect their emotional processing system. Consequently, in moral dilemmas, psychopaths’ tendency to make utilitarian judgments may simply be because their emotional distress will be reduced in hurting another individual. The process-dissociation approach preliminary results reveal the psychological mechanism behind the utilitarian moral judgment of psychopaths.

The experiment results verify the value of the CNI moral model in Chinese culture, which provides more in-depth insight into the contradiction results acquired with the traditional method. Traditional moral dilemmas used in previous studies have indicated that, compared with low psychopathy individuals, high psychopathy individuals are more willing to accept behaviors hurting others in moral dilemmas [27,28,29,30,31,32,33]. In the current study, similar results were found, showing that subjects with high psychopathy symptoms favor making more utilitarian judgments. The premise of utilitarian judgment in the traditional moral dilemma is that it considers the interests of the majority while sacrificing the interests of the minority. Psychopaths who are unlikely to be more concerned about the well-being of most people is not consistent with the behavior patterns of psychopaths. Psychopaths have reduced emotional responses in the amygdala, causing psychopaths compared with non-psychopaths, to hurt others may have less emotionally disgust [39]. Psychopaths who may not be concerned about hurting others in traditional moral dilemmas, unintentional increases the utilitarian choice of psychopaths [27,28,29,30,31,32,33]. Traditional methods do not consider the effect of moral consequences, moral norms, and action response bias on moral judgment. The results of the CNI model analysis show that compared with low psychopaths, people with high psychopathy have low sensitivity to moral norms in Chinese subjects.

Furthermore, those results show that subjects with high psychopathic characteristics and subjects with low psychopathic characteristics show no significant difference in the *C* and *I* parameters. In other words, there was no significant distinction between the two groups regarding their sensitivity to the consequences and their preferred choice for inaction vs. action. However, the study results of Gawronski et al. (2017) have some differences from our study. They point out that high psychopathy subjects have a lower sensitivity to moral consequences and lower sensitivity to moral norms compared to low psychopathy subjects and that their general preference for inaction versus action. The reason for the inconsistent results may be that Gawronski et al. (2017) used the Hare psychopathy scale [24]. In contrast, we used the Chinese version of the LSRP to assess the subjects’ psychopathy symptoms. Additionally, the cultural differences between the East and West are likely another factor. According to Gawronski et al. (2017)’s study, a parameter *I* greater than 0.5 means individuals are not inclined to take action, while a parameter *I* less than 0.5 means individuals are more inclined to take no action [21]. We also found an interesting result that both the high psychopathy and low psychopathy groups in Gawronski et al. (2017)’s research scored greater than 0.5 on the *I* parameter, indicating that Western subjects prefer inaction in CNI moral dilemmas. By contrast, in our study, the *I* parameter of the high psychopathy group was 0.48, and the *I* parameter of the low psychopathy group was 0.49, both less than 0.5, indicating that Chinese subjects prefer action in moral dilemmas. This result may be related to the cultural differences between China and Western countries. People in different countries have different values, which are determined by the social background and culture of their societies. Collectivism is a cultural pattern in most traditional societies, including China [40,41]. It stands in sharp contrast to individualism, a cultural model that exists mainly in North America and Western Europe. Individual independence is the main idea in Western culture, while collectivism is the main idea in Chinese culture [42,43]. These two ideas are formed in the process of social development and are reflected in all aspects of society, thus affecting people’s behavior patterns [44,45]. Collectivism is featured by an emphasis on the cohesion between individuals and the group over the self [46,47]. Western values put special emphasis on hard work and individual rights. Individualists endorse the value of “standing out,” while collectivists often celebrate the individual’s ability to “fit in”. Chinese morals aim at cultivating social and group consciousness and are different from Western individualistic morals [45,47]. Moral judgments are also influenced by cultural values [48,49], and it is this difference in values that may lead to the inconsistency between our study and Gawronski et al.’s study regarding the action preference (*I* parameter).

Moreover, Chinese culture combines individual interests with collective interests and emphasizes a spirit of sacrifice and dedication [48,49]. Confucian culture accentuates the over-emphasis on modern life and daily ethics. The “theory that man is an integral part of nature” in Confucian culture leads to a collectivist orientation and orientation towards the interest of others [50,51,52]. The moral dilemmas create opportunities to save the interests of the majority while sacrificing the interests of the minority. Such a setting may be consistent with the concept of Chinese collectivism, which leads to Chinese subjects being more inclined to take action [52,53]. Generally, these findings further explain the results of previous studies, which also suggest that persons with high psychopathic traits in Chinese culture are more prone to make utilitarian instead of deontological judgments than are low psychopaths.

Our study also has some limitations. First, in this study, prisoners and the clinical population were not included, and the subjects were homogeneous. In future studies, the sample scope could be expanded to further verify the relationship between psychopathy and moral judgment in clinical subjects. Future research can examine the similarities and differences between the clinical population and subclinical psychopathy subjects in moral judgment. Second, we use self-reports to assess psychopathic characteristics and moral judgments and this may be a limitation [54]. Psychopaths are characterized by deception, interpersonal manipulation, and insincerity. We can use implicit association test (IAT) and electrophysiological techniques to further investigate the moral judgment of psychopaths. Furthermore, compared with the traditional moral dilemma paradigm and PD paradigm, the CNI moral model requires a larger sample size, and the use of more experimental dilemma materials is likely to cause subject fatigue. In conclusion, the results of this study can provide some theoretical basis for the psychological intervention treatment of psychopaths through the development of more specific forms of education and intervention employing the cognitive characteristics of psychopaths.

## 5. Conclusions

The CNI moral model analysis is further verification that high psychopaths prefer to make more utilitarian moral judgments. Subclinical psychopaths have a weak sensitivity to moral norms. The sensitivity of the moral consequences of the two groups has no significant difference, as not as the general preference for inaction versus action when faced with moral dilemmas, the moral judgments of high psychopaths do not take more into account the moral consequences in Chinese society.

## Figures and Tables

**Table 1:** The study results between the variables of the mean, standard deviation, and Pearson binary correlation coefficient (*N* = 869).

Variables	*M*(*Mean*)	*SD (Standard Deviation)*	1	2	3	4	5	6
1. Psychopathy	65.49	13.29	1					
2. LSRP-1	37.31	9.77	0.90 **	1				
3. LSRP-2	28.18	6.19	0.73 **	0.35 **	1			
4. Traditional moral judgment (*T*)	4.23	1.33	0.12 **	0.09 **	0.11 **	1		
5. PD utilitarianism (*U*)	0.15	0.23	−0.02	−0.04	0.02	0.53 **	1	
6. PD deontology (*D*)	0.65	0.24	−0.15 **	−0.14 **	−0.09 **	−0.76 **	0.09	1

Note: PD = process dissociation, LSRP-1 = primary psychopathy, and LSRP-2 = secondary psychopathy, ** *p* ≤ 0.01.

**Table 2:** The mean value and 95% confidence interval for the responses to moral dilemmas of action (as opposed to inaction) that include prohibited and prescribed norms and consequences in which the interests of the action are outweighed or less than the losses of action.

Groups	The Prohibited Norms Forbid Action				The Prescribed Norms Prescribe Action			
	Interests of Action Outweigh the Losses		Interests of Action Are Smaller Than the Losses		Interests of Action Outweigh the Losses		Interests of Action Are Smaller Than the Losses	
	*M*	*SD*	*M*	*SD*	*M*	*SD*	*M*	*SD*
Low psychopathy	2.46	1.44	1.56	1.28	4.51	1.26	3.60	1.40
High psychopathy	3.00	1.31	2.18	1.40	3.91	1.46	3.21	1.28

Note: Moral dilemma judgment scores range from 0 to 6. The intermediate reference score for the same number of inaction and action reactions is 3. *M* is the mean, *SD* is the Standard Deviation.
